# Optimization of weak signal propagation in a feedforward network

**DOI:** 10.1186/1471-2202-12-S1-P176

**Published:** 2011-07-18

**Authors:** Muhammet Uzuntarla, Mahmut Ozer, Etem Koklukaya

**Affiliations:** 1 Dept. of Electrical and Electronics Engineering, Zonguldak Karaelmas University, 67100, Zonguldak, Turkey; 2 Department of Electrical and Electronics Engineering, Sakarya University, 54187 Sakarya, Turkey

## 

**Figure 1:**
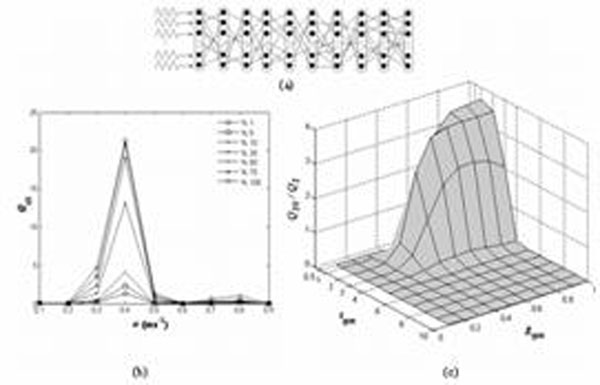
(a) Schematic illustration of a 10 layer feedforward network, (b) Fourier coefficient Q_10_ in dependence on *ω* for different fractions of forced neurons forming the first layer, (c) The dependence of the gain factor Q_10_ / Q_1_with respect to the synaptic transmission parameters.

The ability of weak signal detection and transduction of neurons is of great importance. Although the subject hotly debated in single neuron and complex networks [1], it has been partly addressed in feedforward networks [2,3]. In our previous work [2], we determined the conditions for weak rhythmic signal propagation through a feedforward network where the neurons are constructed with detailed biophysical modeling approaches. It is shown that the optimal propagation of weak rhythmic signals through feedforward neuronal networks depends significantly on the level of intrinsic noise, the forcing frequency and the inter-layer link density. Here, we used the same network structure in [2], which involves 10 layers with N=200 neurons in each one and 10% inter-layer link density (Fig1a). For synaptic transmission, alpha-synapse type is used to model the conductance variations in the post-synaptic neuron resulting from the binding of neurotransmitter released from the pre-synaptic neuron. Release time of neurotransmitters τ_syn_ and coupling constant g_syn_ are critical parameters of this type of synapses. A measure Q_i_ is chosen to quantify the signal transmission efficiency, which gives the existence of the input signal frequency at any layers’ output, similar to Signal to Noise Ratio (SNR). Model details and methods can be found in [2].

To extend our findings, we first investigated the role of fraction of forced neurons forming the first layer on the propagation of weak rhythmic activity. We consider different fractions of periodically forced neurons (chosen randomly) and compute Q_10_ (Fourier coefficients of output layer) over a broad frequency range (Fig 1b). Evidently, the larger the fraction of neurons subject to the weak forcing, the better the outreach of the signal through the network. This can be appreciated most clearly for the optimal angular forcing frequency, *ω*≈**0.4ms**^–1^[2]. Interestingly, the depicted curves show only marginal improvement in Q_10_ if more than 50% of the neurons forming the first layer are forced. This finding suggests that the propagation of weak signals across feedforward neuronal networks is rather robust to variations in the coverage of the initial input.

We also examined how synaptic mechanisms effect the propagation of weak rhythmic activity through the network. For this purpose, a periodic sinusoidal force with the optimal angular frequency *ω*≈**0.4ms**^–1^ is introduced to all neurons in the first layer as shown in Fig1a, then we computed *Q_10_*/*Q_1_*gain factor with respect to systematically changed values of synaptic parameters. Results are presented in Fig1c. It is clearly seen that signal transmission can be modulated by the synaptic transmission parameters*.* There is an optimal *τ_syn_* range, i.e. 1.5 <τ_syn_ < 4ms , where the gain factor is switched from Q_10_ / Q_1_ <1 to Q_10_ / Q_1_>=1 for g_syn_ >0.4 nS, indicating the efficient transmission of the weak signal. The underlying mechanism can be understood by comparing synaptic and membrane time constants with respect to each other, because they together determine the operation mode of neurons, i.e. coincidence detector or temporal integrator [3]. Fig 1c also shows that weak signal can be transmitted to the output layer for the coupling constants of g_syn_ >0.4 nS regardless ofτ_syn_, implying that the synaptic coupling must be higher than a critical value.
